# A Comprehensive In Vitro Assessment of Broad-Spectrum Antimicrobial Efficacy of Organo-Selenium-Incorporated Urinary Catheter in Comparison to a Zwitterionic Surface Catheter

**DOI:** 10.3390/antibiotics15060574

**Published:** 2026-06-04

**Authors:** Harry May, Md Abid Afridi, Phat L. Tran, Hannah Seo, Eric Tran, Wei Li, Ted W. Reid, Werner T. W. de Riese

**Affiliations:** 1 School of Medicine, Texas Tech University Health Sciences Center, Lubbock, TX 79430, USA; harry.may@ttuhsc.edu (H.M.); hannah.seo@ttuhsc.edu (H.S.); eric1.tran@ttuhsc.edu (E.T.); 2 Department of Chemical Engineering, Texas Tech University, Lubbock, TX 79409, USAwei.li@ttu.edu (W.L.); 3 Department of Ophthalmology and Visual Sciences, Texas Tech University Health Sciences Center, Lubbock, TX 79430, USA; phat.tran@ttuhsc.edu; 4 Department of Urology, Texas Tech University Health Sciences Center, Lubbock, TX 79430, USA; werner.deriese@ttuhsc.edu

**Keywords:** catheter-associated urinary tract infection, uropathogenic microbial spectrum, antimicrobial materials, selenium, zwitterionic coating, in vitro growth inhibition

## Abstract

**Background/Objectives**: Catheter-associated urinary tract infections (CAUTIs) are one of the most common healthcare-related morbidities. Also, severe clinical outcomes derived from CAUTIs demand an urgent need for the development of novel antimicrobial catheter materials. Since CAUTIs are primarily driven by a wide range of microorganisms causing biofilm formation on the surfaces of catheters, evaluating the effectiveness of innovative antimicrobial materials against a broad spectrum of known uropathogens is warranted. We aim (1) to demonstrate the ability of incorporated organo-selenium versus that of an FDA-cleared antimicrobial catheter and (2) to show that the results of the study are consistent against the most common microorganisms causing urinary tract infections in humans. **Methods**: Based on encouraging preliminary studies, three percent of organo-selenium (weight-based), as a novel antimicrobial catheter material, was incorporated into thermoplastic polyurethane (TPU). This was compared in vitro with plain polyurethane catheters and the SILQ ClearTract catheter with a zwitterionic coating (FDA-cleared for its antimicrobial properties in December 2022). The antimicrobial activity was studied against *Candida albicans*, *Escherichia coli*, *Klebsiella pneumoniae*, *Proteus mirabilis*, *Pseudomonas aeruginosa*, *Staphylococcus aureus*, MRSA, and *Staphylococcus epidermidis* by assessment of colony-forming unit counts along with visual confirmation using Scanning Electron Microscopy (SEM). **Results**: Plain polyurethane catheters (control group) showed 7- to 8-log of in vitro growth for all tested microbes, whereas the antimicrobial zwitterionic SILQ ClearTract catheters still showed 6- to 7-log of microbial growth. In contrast, organo-selenium-incorporated catheters demonstrated no detectable in vitro growth for all tested microbes (*C. albicans*, *E. coli*, *K. pneumoniae*, *P. mirabilis*, *P. aeruginosa*, *S. aureus*, MRSA, and *S. epidermidis*). SEM analysis also validated the findings. **Conclusions**: Potentially non-leaching organo-selenium, as a novel urinary catheter material, significantly inhibited microbial attachment, growth, and biofilm formation across a wide spectrum of common uropathogenic organisms compared to a zwitterionic catheter, providing a strong foundation for further detailed in vivo and clinical studies.

## 1. Introduction

Catheter-associated urinary tract infections (CAUTIs) are one of the most common healthcare-related infections and are reported for 70–80% of all nosocomial urinary tract infections [1]. Between 15% and 25% of hospitalized patients require a catheter during their admission, making CAUTIs a complication of inpatient care that could be prevented [2]. These infections tend to be associated with prolonged hospital stays and increased morbidity, which require substantial healthcare resources. One million cases are estimated annually only in the United States, with the estimated cost around $1.8–2 billion annually [3]. In addition, 2–4% of CAUTIs progress to bacteremia and even severe sepsis, which leads to a three-fold increase in mortality compared with non-bacteremic patients [4].

CAUTIs are primarily driven by microbial adherence to the surfaces of catheters, which leads to biofilm formation. Biofilms are made up of complex microbial communities encased in an extracellular polymeric matrix that creates shields from the immune system and resistance to antimicrobial treatment [5]. The organisms in CAUTIs frequently include *Escherichia coli*, *Staphylococcus aureus*, *Klebsiella pneumoniae*, *Haemophilus influenzae*, *Pseudomonas aeruginosa*, and *Candida albicans* [5]. Even with a fundamental understanding of CAUTI mechanisms and recent progress on prevention strategies, which have decreased prevalence by roughly 10%, more than 560,000 patients in the U.S. still develop a CAUTI each year [6]. Accompanying this high incidence, complications arising from urinary catheter use persist. Consequently, the development of effective antimicrobial surfaces on urinary catheters is needed to mitigate infection risks and improve patient outcomes.

**Figure 1:**
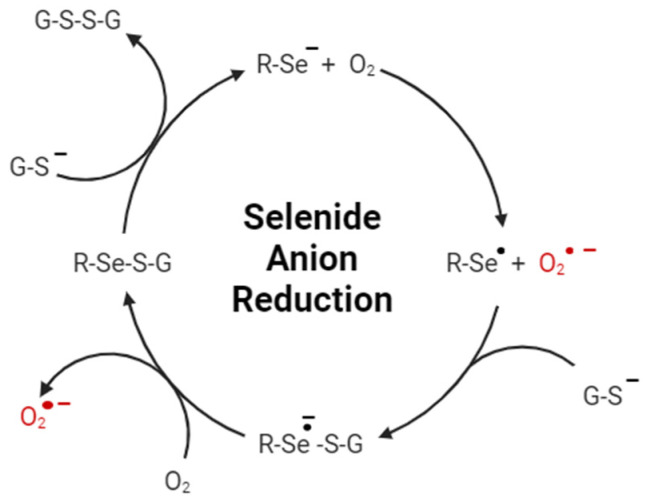
Equation of the selenide anion reduction pathway and generation of superoxide.

Organo-selenium brings a different antimicrobial mechanism. It catalyzes the formation of superoxide radicals via redox cycling with sulfur compounds, including glutathione, which is universally present in biological tissues [7] (Figure 1). These formed radicals cause oxidative stress to exposed bacterial cells, which leads to cell death. These superoxide radicals cause minimal toxicity to the surrounding tissue due to their short-lived characteristics. Their excellent biocompatibility with surrounding human tissues made organo-selenium one of the best candidates to combat CAUTI. Studies on organo-selenium-treated medical devices, such as contact lenses, tooth sealants, and hemodialysis catheters, have validated the antimicrobial efficacy and biocompatibility of organo-selenium [8,9,10].

A previous study revealed that 1% organo-selenium incorporated catheters demonstrated complete growth inhibition of *E. coli*, *K. pneumoniae*, and *H. influenzae*, while *P. aeruginosa* was inhibited by approximately 99.99% [11]. Another study conducted dose–response investigations and revealed that at 2.5% selenium, *P. aeruginosa* showed no growth; furthermore, *C. albicans* and *S. aureus* were fully stopped even at 1% selenium [7]. These in vitro data suggested that organo-selenium catheters possessed a marked antimicrobial potency against the aforementioned CAUTI-associated pathogens, compared to the reported in vitro inhibitory activity of coated catheters with nitrofurazone or silver-alloy. In December 2022, the SILQ ClearTract catheters, manufactured with a zwitterionic-coated technique, achieved FDA clearance for their antimicrobial features [12].

Here, the presented study directly compares the antimicrobial characteristics of polyurethane catheters and the zwitterionic-coated SILQ ClearTract catheters, both FDA approved/cleared, with the newly developed selenium-incorporated catheters (not yet FDA-approved).

## 2. Results

### 2.1. Differential Antimicrobial Efficacy of Zwitterionic and Organo-Selenium Coatings

**Figure 2:**
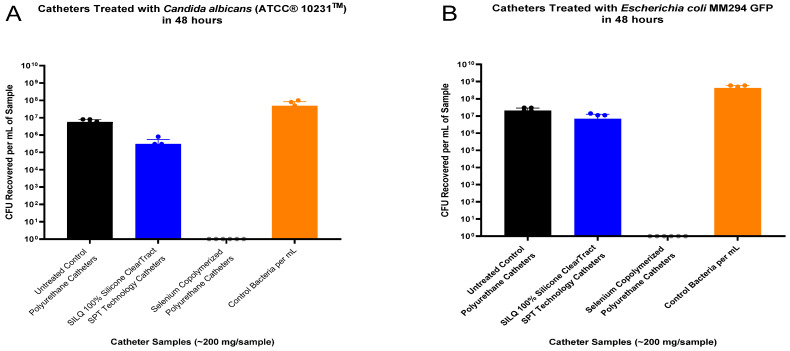

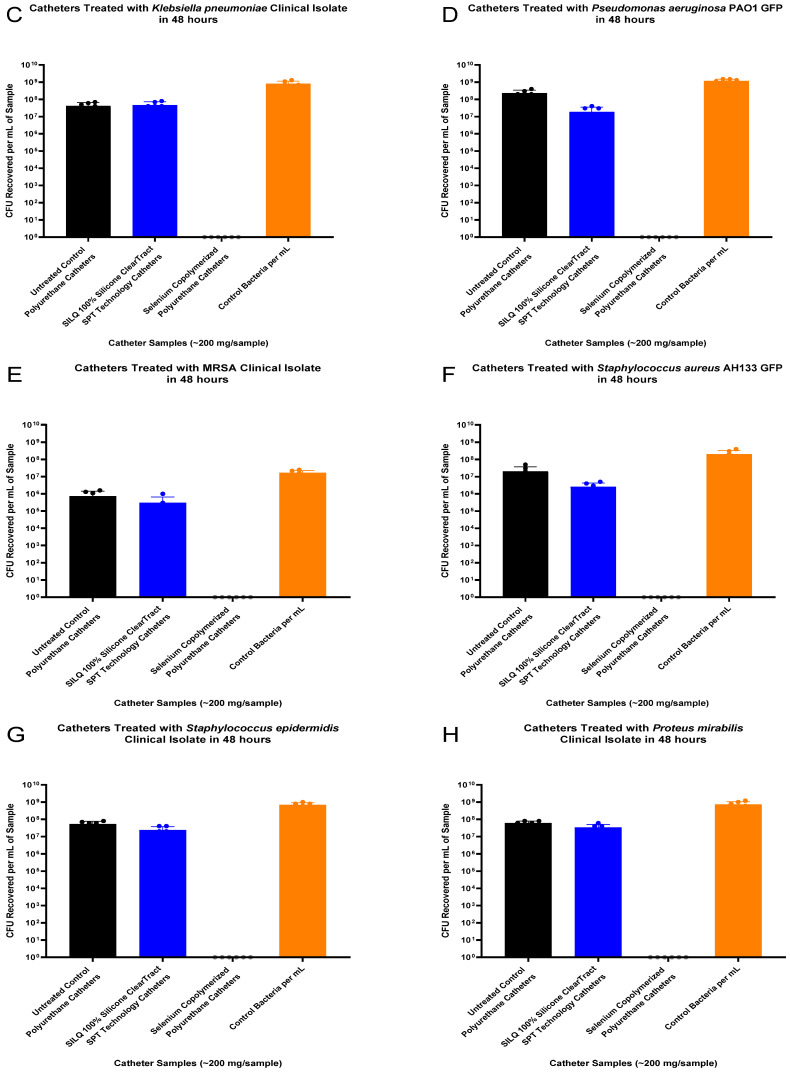
Growth of different uropathogenic agents on TPU catheter, SILQ ClearTract zwitterionic catheter, and organo-selenium-incorporated catheter. (**A**) *Candida albicans*, (**B**) *Escherichia coli*, (**C**) *Klebsiella pneumoniae*, (**D**) *Pseudomonas aeruginosa*, (**E**) MRSA, (**F**) *Staphylococcus aureus*, (**G**) *Staphylococcus epidermidis*, and (**H**) *Proteus mirabilis*.

Following a 48 h incubation of bacterial and fungal uropathogens, CFU quantification revealed that, compared to control TPU catheters, SILQ ClearTract catheters reduced the growth of *C. albicans* (by ~2-log), *E. coli* (~1/2-log), *P. mirabilis* (~1/2-log), *P. aeruginosa* (~1-log), *S. aureus* (~1-log), and *S. epidermidis* (~1/2-log) (Figure 2). However, they did not yield a statistically significant growth inhibition of *K. pneumoniae* and MRSA. In comparison to the SILQ ClearTract catheters, the organo-selenium-incorporated catheters demonstrated superior efficacy in inhibiting the growth across all tested uropathogens by 6-log (*C. albicans*), 7-log (*E. coli*), 7-log (*K. pneumoniae*), 7-log (*P. mirabilis)*, 8-log (*P. aeruginosa)*, 6-log (MRSA), 7-log (*S. aureus*), and 7-log (*S. epidermidis)* reduction. One-way ANOVA showed that, in terms of inhibitory effects, the SILQ catheter achieved high significance (*p* < 0.0001) against agents *C. albicans* and *P. aeruginosa*. However, it exhibited lower or non-statistically significant activity against *E. coli* (*p* = 0.006), *K. pneumoniae* (ns), *P. mirabilis* (*p* = 0.03), MRSA (ns), *S. aureus* (*p* = 0.0006), and *S. epidermidis* (*p* = 0.01). The SILQ catheter did not match the performance against the organo-selenium-containing catheter, which achieved significant inhibition against all tested uropathogens.

### 2.2. Comparative SEM Analysis of Distinctive Antimicrobial Activities

**Figure 3:**
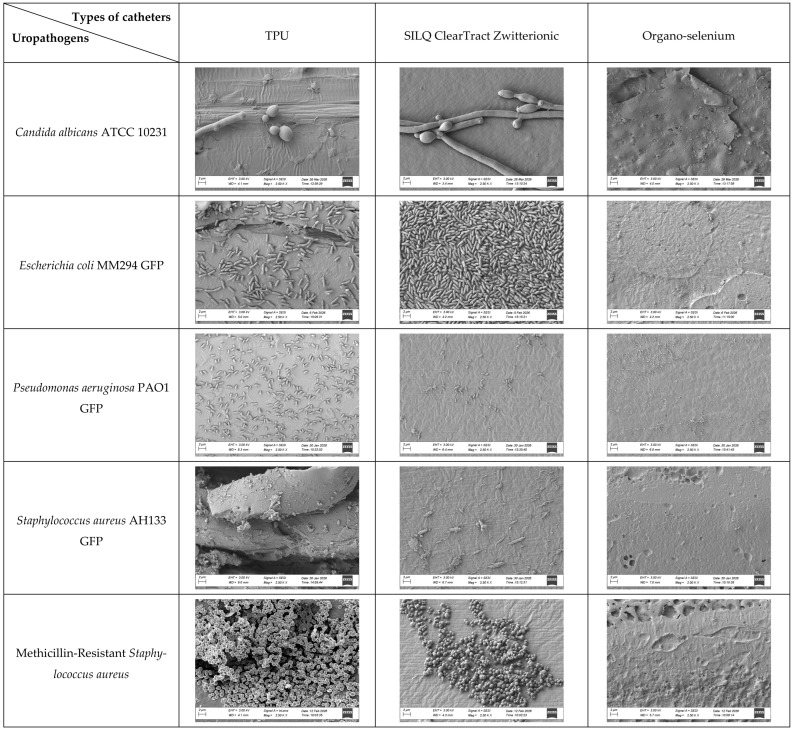

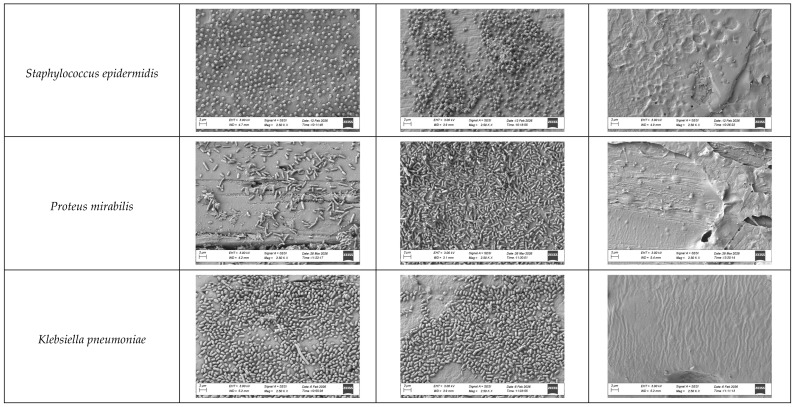
Growth on the **external** surface of each catheter type by SEM.

**Figure 4:**
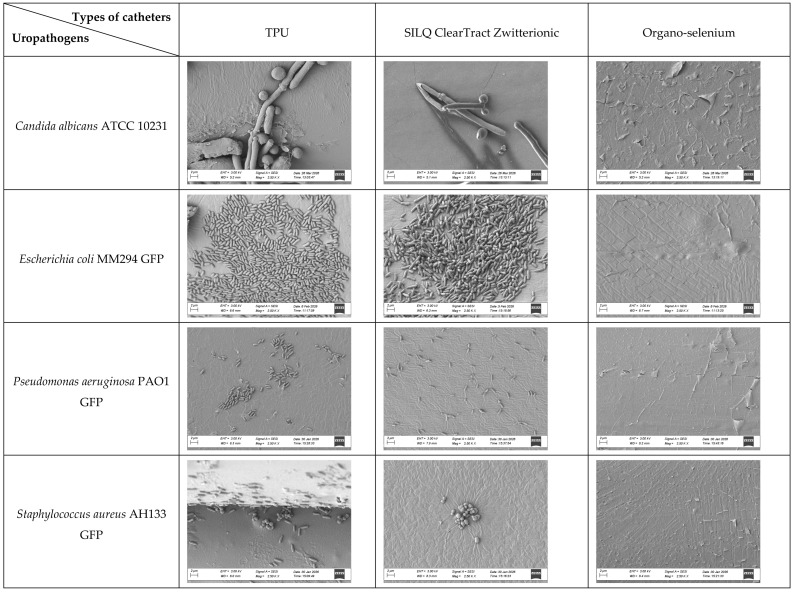

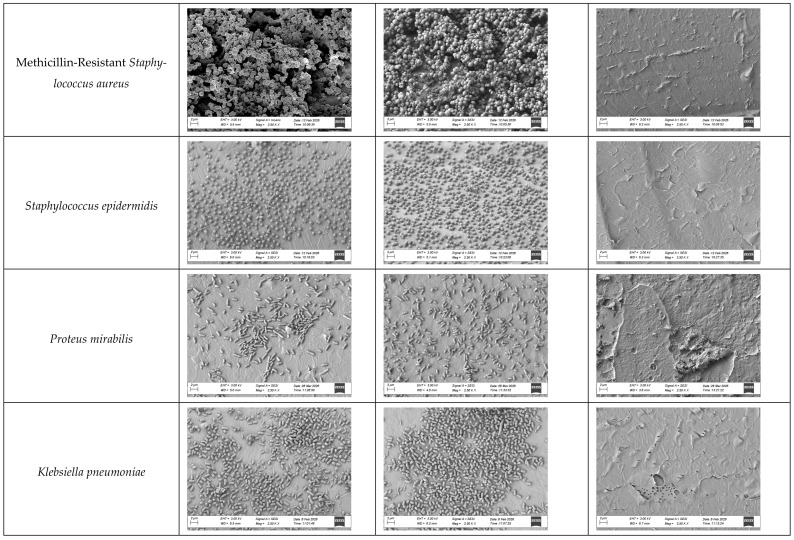
Growth on the **internal** surface of each catheter type by SEM.

Given that the pathogenesis of CAUTI includes extraluminal and intraluminal routes, assessment of the antimicrobial activities on both external and internal catheter surfaces is necessary. Images from the external surface of catheters validated the differential growth inhibition by CFU counting (Figure 3). While SILQ ClearTract catheters significantly decreased the external surface colonization of *C. albicans*, *P. aeruginosa*, and *S. aureus*, the colonization levels of *E. coli*, *K. pneumoniae*, *P. mirabilis*, MRSA, and *S. epidermidis* remained visually comparable to those of control TPU catheters. The colonization inside the catheter surface showed similar results (Figure 4). In contrast, organo-selenium-incorporated catheters demonstrated a complete inhibition of microbial colonization on both inside and outside the catheter surface.

### 2.3. Planktonic Growth Inhibition by Organo-Selenium Catheter

**Figure 5:**
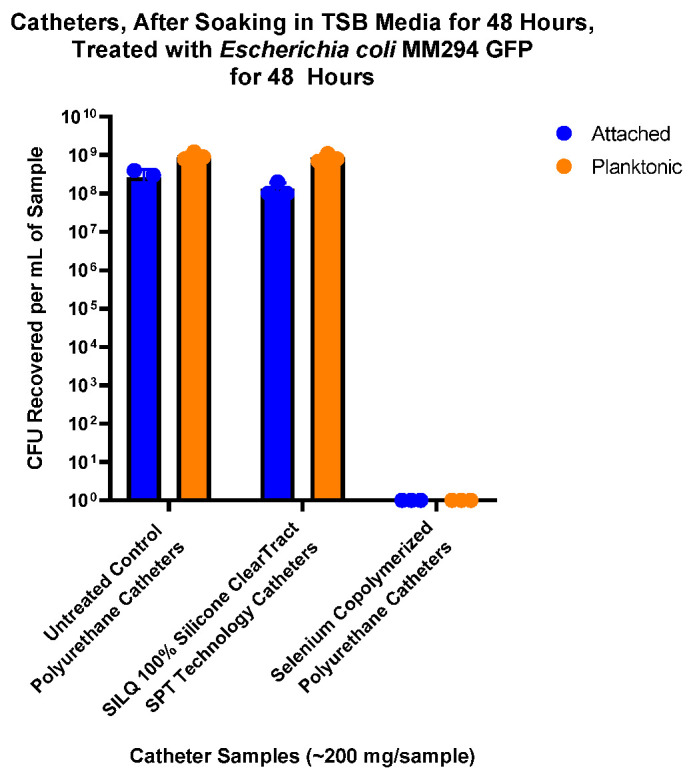
Planktonic growth inhibition of *E. coli* MM294 by organo-selenium catheters.

Planktonic cells within urinary catheters can colonize the internal surface, resulting in biofilm formation. Conversely, urinary catheter colonization can be facilitated by dispersed release of planktonic cells from existing biofilms. We performed the CFU assay to confirm the bactericidal activity of surface-level superoxide radicals against planktonic cells. Also, catheters were soaked in TSB media for 48 h prior to incubation with *E. coli* to detect a reduced bactericidal efficacy due to a potential leaching effect. Figure 5 demonstrated that the bactericidal activity of the organo-selenium catheter against planktonic cells showed growth reduction in both attached and planktonic cells in comparison to SILQ ClearTract catheters (both *p* < 0.0001) after 48 h incubation of TSB, suggesting the bactericidal efficacy of the organo-selenium catheters with potentially negligible leaching effects.

## 3. Discussion

Despite many efforts, the healthcare environment still faces significant challenges due to the rising incidence of multidrug-resistant CAUTI. A clinical study confirmed that while 92% of CAUTIs are resistant to at least one common antibiotic substance, approximately 80% are resistant to multiple agents [13]. This significantly compromises patient outcomes as patients with drug-resistant UTIs are prone to prolonged hospital stays and, thus, higher medical costs. They are also twice as likely to experience complications such as recurrent severe infections and sepsis, which lead to increased mortality [14,15]. These documented clinical trends in humans underscore the need for developing and manufacturing catheters with excellent broad-spectrum antimicrobial features.

An effective way to combat CAUTI includes compliance with rigorous catheter maintenance as well as avoiding any blockage of urine flow. The risk of bacteriuria in patients with indwelling bladder catheters increases by 3% to 10% daily, reaching a total of 100% after 3–4 weeks; thus, long-term use is generally discouraged [16]. However, there are many clinical circumstances where long-term catheter use is unavoidable, in particular for patients with chronic urinary retention or urinary incontinence, as often seen in nursing homes [17]. Despite numerous efforts to lower the risks for CAUTI, there is a real clinical need for the development of improved antimicrobial urinary catheter materials that can safely accommodate extended catheter use [18].

Several emerging catheter manufacturing methods have been proposed and undergone clinical trials to address infection risks. Key advancements focus on catheter coatings either to inhibit bacterial attachment and ultimately biofilm formation, or to directly kill pathogens. The SILQ ClearTract catheter offers a unique, FDA-cleared alternative to traditional drug-eluting devices for urethral, suprapubic, and nephrostomy use. Utilizing a zwitterionic coating rather than antibiotics or silver alloys creates a permanent, microscopic “water shield” around the catheter, preventing bacteria from sticking to the catheter material and thereby preventing the early stage of biofilm formation [12].

While many novel catheters have received FDA clearance, none of them have ever become the standard of care. The CDC currently recommends using antimicrobial-impregnated catheters only if standard infection prevention strategies, such as aseptic insertion and compliant maintenance, have failed to reduce a facility’s CAUTI rate [1]. The main reasons for this recommendation are: (1) A clinical trial in the UK demonstrated that antimicrobial catheters, such as silver alloy catheters, compared to controls, showed no significant advantages in preventing CAUTI even during short-term use [19]; (2) economic analysis of a clinical trial showed that the higher costs of novel antimicrobial catheters made them less cost-effective when compared to traditional catheters [20]; and (3) antimicrobial-coated catheters were still susceptible to biofilm formation over extended periods, such as 3–4 weeks [21].

In terms of prevention of CAUTIs caused by long-term use, selenium-coated catheters offer several distinct advantages over current FDA-cleared antimicrobial options. First, in contrast to silver alloy coatings for urinary catheters, selenium is a naturally occurring trace element essential for human health, playing a crucial role in thyroid hormone metabolism, DNA production, and antioxidant defense against cell damage [22]. The low selenium concentration shown in this study did not cause any adverse toxic effects on human mucosal surfaces, as demonstrated with FDA-approved tooth sealants and tooth braces [7]. Second, the covalent polymerization of organo-selenium into the catheter matrix effectively eliminates the issue of agent leaching, a major disadvantage of silver- or antibiotic-coated catheters, achieving a significant growth reduction even after long-term (12 weeks) incubation in PBS as comparable as in the short-term [11]. Furthermore, as their agent concentration depletes on the catheter surface over time due to the leaching effect, bacteria often develop resistance to antibiotic-coated catheters, as seen with antibiotics given PO or IV, as well as with silver-coated catheters [23]. Since the selenium compound could be directly polymerized in the polyurethane catheter material, there is no leaching effect over time, thus maintaining the therapeutic concentration of surface-level superoxide radicals to effectively kill pathogens on contact. Third, while a study on silver-alloy latex hydrogel catheters showed a reduced adherence of *E. coli* to the catheter surface only, the selenium compound in the presented study showed a near-complete inhibition of bacterial and fungal growth [24]. Likely related to the leaching effect of silver and antibiotic-coated catheters, clinical studies revealed the poor efficacy of silicon-based coated catheters in long-term use [25]. Therefore, selenium provides a more durable antimicrobial effect. Finally, while several coated catheters have been introduced, long-term clinical data are still needed to determine if a potential reduction in CAUTI rates and subsequent healthcare resource utilization is sufficient to justify their higher manufacturing cost [26]. However, the selenium compound could be a cost-effective alternative. A previous study on the long-term antimicrobial features of the selenium compound after soaking the catheters in PBS for 12 weeks, followed by inoculation with *E. coli*, still showed steady and excellent antimicrobial effectiveness, suggesting that their clinical benefits may mitigate the manufacturing costs, specifically in the setting of long-term use [11].

Here, our in vitro study confirmed that selenium catheters demonstrated superior antimicrobial activity to catheters with a zwitterionic coating. The SILQ catheters showed less statistically significant growth inhibition for *E. coli*, *K. pneumoniae*, *P. mirabilis*, MRSA, *S. aureus*, and *S. epidermis*, compared to selenium catheters that demonstrated near-complete growth inhibition for all eight studied microorganisms, including these six microorganisms. Selenium also showed significantly better in vitro results against *Proteus* than the SILQ catheters. *Proteus* is a pathogen responsible for about 40% of CAUTIs, particularly in patients with long-term catheter use, suggesting that organo-selenium incorporated catheters could be an effective long-term method for preventing CAUTIs [27]. Moreover, the bacterial and fungal species tested in this report represent the four most prevalent CAUTI pathogens [28]. Given that up to 86% of CAUTIs involve multiple microbial species, the broad-spectrum antimicrobial activity of selenium-incorporated catheters makes them a compelling alternative to current options [29].

The SILQ catheter showed decreased CFU values but not close to complete in vitro growth inhibition when compared to the control/uncoated polyurethane catheter in our study. These results are best explained by its anti-adhesive effect against microbial attachment, but apparently, this catheter material has limitations, potentially due to its lack of bactericidal effects. The lack of growth inhibition in *K. pneumoniae* and MRSA may stem from distinct survival mechanisms that allow these pathogens to overcome the anti-adhesive effect of zwitterionic coatings. Beyond its efficacy in total biomass reduction and inhibition of average biofilm thickness, it provides superoxide radicals with direct cytotoxic effects on microbes [30]. Thus, the development of any bacterial or fungal resistance is unlikely even during long-term use.

This study expands the knowledge on selenium-incorporated catheters beyond previous studies by testing a broader range of CAUTI pathogens and providing a direct comparison with the FDA-cleared SILQ catheter. In addition to predominant CAUTI-causing agents, this study also included *S. epidermidis* and MRSA. Even though they are not common causes of CAUTI, MRSA dissemination to bacteremia is a serious and feared complication of CAUTI, which occurs more frequently and develops more rapidly compared to other CAUTI-causing agents (20% vs. 5%) [31]. Our study results revealed that organo-selenium achieved near-complete growth inhibition of these high-risk bacteria, whereas the SILQ catheter only showed a partial or no growth reduction. These results correspond well to a study on selenium nanoparticles showing strong biofilm prevention for *S. epidermidis* and MRSA on orthopedic implants [32]. The unmatched total growth inhibition of both *S. epidermidis* and MRSA by selenium suggests the universal adoption of selenium catheters in preventing life-threatening systemic complications of CAUTIs.

While the results of this study are impressive for selenium-incorporated catheters, we also see the following main limitations in our study. Selenium-incorporated catheters are currently in the early experimental stages. Even though the in vitro results have been consistent and correspond well with previously reported findings and direct catheter surface visualization by SEM, additional in vivo/animal data are necessary to confirm its efficacy and reproducibility, which may then lead to clinical trials. Low-dose selenium has been proven in clinical trials to be well-tolerated and nontoxic in patients treated with tooth sealing and braces. These results suggest favorable outcomes in clinical selenium catheter trials with minimal adverse effects. We are currently establishing a robust animal/pig model for CAUTI. This will enable a direct in vivo comparison between selenium-incorporated and control catheters against *E. coli*, the most prevalent cause of CAUTI. Based on the study results and future replicable in vivo experimental outcomes, clinical trials in humans may be feasible and justified.

## 4. Materials and Methods

### 4.1. Materials

Thermoplastic polyurethane (TPU) filament was purchased from Overture (Houston, TX, USA). Dimethyl sulfoxide (DMSO, 99.9%, ACS grade) was obtained from Fisher Chemical. Toluene diisocyanate (TDI) mixture of isomers for synthesis was obtained from Sigma-Aldrich (St. Louis, MO, USA). Selenium-containing compounds, C_3_O_2_H_8_Se (51.3% Se) and C_4_O_2_H_10_Se (47.0% Se), were used as received.

### 4.2. Filament Preparation

**Figure 6:**
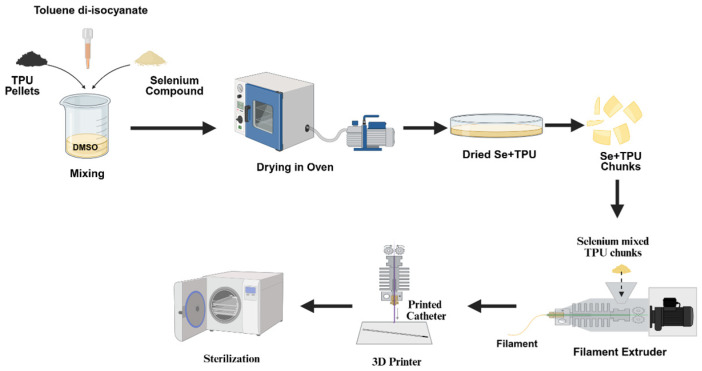
Schematic illustration of TPU-Se composite catheter fabrication process.

The comprehensive schematic illustration is presented in Figure 6. Thermoplastic polyurethane–selenium (TPU-Se) composites were synthesized through solution blending, solvent evaporation, and melt extrusion. A total of 97 g TPU was dissolved in DMSO at a 1:1 (*w*/*w*) ratio in sealed glass beakers and maintained at room temperature for 24 h to yield a viscous, homogeneous solution. A total of 3 g of the selenium compound was dissolved separately in DMSO at a 1:1 (*w*/*w*) ratio to form a concentrated selenium solution. This selenium solution was incorporated into the TPU solution with manual mixing to ensure uniform distribution. Subsequently, 50 g of DMSO and 1 mL of TDI were added, and the mixture was manually homogenized.

The prepared solution was transferred to an oven and heated at 70 °C for 48 h to facilitate the removal of DMSO. To minimize phase separation and promote compositional uniformity, the mixture was manually stirred every 6 h during solvent evaporation. After 30 h, the highly viscous solution was transferred to an antistatic thin polystyrene plate (for easy removal) and then returned to the oven at 70 °C for an additional 18 h of solvent evaporation. After complete solvent removal, the material was extracted from the polystyrene dish and thermally treated at 150 °C for 24 h to obtain a solid TPU-Se composite. The bulk composite was then cut into small pieces and melt-extruded at 200 °C to produce 1.75 mm-diameter TPU-Se filaments suitable for fused deposition modeling (FDM). Control (non-selenium) samples were prepared by dissolving TPU in DMSO (1:1, *w*/*w*) and applying the same solvent evaporation and thermal-treatment protocol.

### 4.3. In Vitro Plate Preparation

For in vitro testing, 15 g of the homogeneous TPU-Se-DMSO-TDI mixture was cast into a 100 mL antistatic thin polystyrene plate (89 × 89 × 25 mm) and subjected to the same solvent evaporation (80 °C, 48 h with intermittent mixing) and then transferred to a glass Petri dish for post-curing (150 °C, 24 h), as described above to produce solid composite plates. Control plates without selenium were fabricated using an identical procedure.

### 4.4. Device Design and Fabrication

Urinary catheter models (14 Fr and 16 Fr) were designed using Rhinoceros 3D software v8.21 with a uniform wall thickness of 0.85 mm. The distal end of each catheter incorporated a spiral geometry integrated with the main body to ensure compatibility with a Luer-lock syringe (Becton, Dickinson and Company, Franklin Lakes, NJ, USA) for fluid collection. Designs were exported as STL files, converted to G-code using Creality Slicer, and fabricated on a Creality Ender-3 V3 KE 3D Printer, 500 mm/s High-Speed Printer (Shenzhen Creality 3D Technology Co., Ltd., Shenzhen, China). Printing parameters were set to 210 °C, 10 mm s^−1^, a gyroid infill pattern, and a layer height of 0.12 mm. The printed samples were polished with sandpaper to smooth the surface and then sonicated in a bath sonicator to remove the debris. Samples were washed simultaneously in ethanol and distilled water, then dried at 50 °C in an oven for 1 h. Finally, the samples were autoclaved in autoclave bags at 121 °C for 15 min.

### 4.5. Growth Conditions, Bacterial Strains, and Media

*Escherichia coli* GFP strain MM294, which constitutively expresses green fluorescent protein (GFP) from plasmids pCM11 and pMRP9-1, respectively, was used along with *Pseudomonas aeruginosa* PAO1 GFP and *Staphylococcus aureus* AH133 GFP. *Klebsiella pneumoniae*, *Proteus mirabilis*, Methicillin-Resistant *Staphylococcus aureus*, and *Staphylococcus epidermidis* clinical isolates at our institution were also used. *Candida albicans* strain 3147 (ATCC 10231) was also included in this study. The strains were routinely grown in Luria–Bertani broth (LB) or Yeast Extract Peptone Dextrose (YPD) at 37 °C with shaking (250 rpm). The pCM11 was maintained in AH133 using both LB and trypticase soy broth (TSB) supplemented with 1 μg/mL erythromycin. To maintain pMRP9-1 in MM294, both LB and TSB were supplemented with 300 µg/mL carbenicillin.

### 4.6. Scanning Electron Microscopy

The procedure was described previously [11]. Following a 48 h incubation at 37 °C, the samples were washed three times with Phosphate-Buffered Saline (1X PBS, pH = 7.4) to remove planktonic microorganisms. Any attached media was gently wiped and shaken off the samples. The samples were then fixed in 2.5% glutaraldehyde. The glutaraldehyde-fixed samples were washed every 10 min with 0.05 M cacodylate buffer three times to completely remove the residual. Then, the samples were soaked in 0.1% OsO4 in 0.05 M cacodylate solution for 30 min for postfixation. After washing with cacodylate buffer every 10 min three times, the graded ethanols at 25%, 50%, 75%, 85%, 95%, and 100% were used to dehydrate the samples at 10 min intervals. The samples were then dried by a critical point dryer, followed by mounting onto the sample mounts, and coated with a thin layer of Ir for conductivity. The images were taken by a Zeiss crossbeam 540 microscope at 3 kV and 2.5 k x magnification with a secondary electron detector (Carl Zeiss Microscopy GmbH, Jena, German).

### 4.7. Colony Forming Unit (CFU) Determination

This procedure was previously described [7]. Six catheter pieces (1 cm long and 3 mm in diameter) of the control TPU or SILQ ClearTract catheter or TPU-Se were placed into individual wells of sterile Falcon 24-well polystyrene plates (Becton Dickinson Labware, Bedford, MA, USA). Each well contained 1 mL of TSB inoculated with approximately 10^3^ to 10^4^ CFU of microorganisms. Three wells containing TSB only were used as negative controls. The plates were covered and incubated at 37 °C for 48 h with slight shaking on a Titer Plate Shaker (Lab-line Instruments; Melrose Park, IL, USA). For *Candida albicans* strain 3147 (ATCC 10231), 10^3^ to 10^4^ CFU in one mL of YPD (yeast extract peptone dextrose) were inoculated into each well and incubated at 37 °C for 48 h. Three wells containing only YPD were used as negative controls. To determine the CFUs, the six pieces were carefully removed from each well of the 24-well plates with sterile forceps, rinsed several times gently with 1X PBS, and placed into a 1.5 mL micro-centrifuge tube containing 1 mL of 1X PBS. The tubes were vigorously vortexed three times, for 2–3 min each time, to disrupt the biofilm and detach the bacteria/fungus from the pieces. Suspended cells were then serially diluted (10-fold) in 1X PBS, and 10 µL aliquots of each dilution were spotted on YPD agar plates for *Candida albicans* or on LB agar plates for *Escherichia coli*, *Klebsiella pneumoniae*, *Proteus mirabilis*, *Pseudomonas aeruginosa*, *Staphylococcus aureus*, Methicillin-Resistant *Staphylococcus aureus*, and *Staphylococcus epidermidis*. The plates were incubated at 37 °C for 24 h, and the number of CFUs was counted. The number of CFU per piece was determined using the following formula: CFU counted × dilution factor × 200. The experiments were performed in sextuplicate. For planktonic cell measurement, the catheters were soaked in TSB media for 48 h at room temperature. The catheters were then used for testing *E. coli*.

### 4.8. Graphing and Statistical Analyses

CFU assay data were analyzed and graphed using GraphPad Prism 4.03 (GraphPad Software, San Diego, CA, USA). All experimental procedures were performed in sextuplicate to ensure reproducibility. Due to the exponential nature of the high range of the data, values were log-transformed prior to analysis. Both Shapiro–Wilk tests and Q-Q plots revealed that the data satisfied the assumptions for parametric analysis, one-way ANOVA. Statistical significance was determined as *p* < 0.05.

## 5. Conclusions

CAUTIs remain a major source of morbidity, mortality, and financial burden, specifically in the settings of increasing incidence of multidrug resistance with long-term catheterization. The presented in vitro data show that potentially non-leaching organo-selenium-incorporated catheters outperform FDA-cleared SILQ catheters; thus, the use of organo-selenium-incorporated catheters offers a promising alternative for future CAUTI prevention through its effective growth inhibition across a broad range of CAUTI pathogens. Taken together, these encouraging findings justify proceeding with animal studies and, eventually, human clinical trials.

## Data Availability

Data are contained within the article.

## References

[1] Patel P.K., Advani S.D., Kofman A.D., Lo E., Maragakis L.L., Pegues D.A., Pettis A.M., Saint S., Trautner B., Yokoe D.S. (2023). Strategies to prevent catheter-associated urinary tract infections in acute-care hospitals: 2022 Update. Infect. Control Hosp. Epidemiol..

[2] Clarke K., Hall C.L., Wiley Z., Tejedor S.C., Kim J.S., Reif L., Witt L., Jacob J.T. (2020). Catheter-Associated Urinary Tract Infections in Adults: Diagnosis, Treatment, and Prevention. J. Hosp. Med..

[3] Werneburg G.T. (2022). Catheter-Associated Urinary Tract Infections: Current Challenges and Future Prospects. Res. Rep. Urol..

[4] Stamm W.E. (1991). Catheter-associated urinary tract infections: Epidemiology, pathogenesis, and prevention. Am. J. Med..

[5] Tegegne D.T., Abbott I.J., Poźniak B. (2025). Catheter-Associated Urinary Tract Infections: Understanding the Interplay Between Bacterial Biofilm and Antimicrobial Resistance. Int. J. Mol. Sci..

[6] Gould C.V., Umscheid C.A., Agarwal R.K., Kuntz G., Pegues D.A., Healthcare Infection Control Practices Advisory Committee (HICPAC) (2010). Guideline for Prevention of Catheter-Associated Urinary Tract Infections 2009. Infect. Control Hosp. Epidemiol..

[7] Tran P.L., Presson C.L., Kashem M.N.H., Li W., Reid T.W., De Riese W.T.W. (2024). Complete Growth Inhibition of Pseudomonas aeruginosa by Organo-Selenium-Incorporated Urinary Catheter Material. Antibiotics.

[8] Mathews S.M., Spallholz J.E., Grimson M.J., Dubielzig R.R., Gray T., Reid T.W. (2006). Prevention of Bacterial Colonization of Contact Lenses with Covalently Attached Selenium and Effects on the Rabbit Cornea. Cornea.

[9] Tran P.L., Lowry N., Campbell T., Reid T.W., Webster D.R., Tobin E., Aslani A., Mosley T., Dertien J., Colmer-Hamood J.A. (2012). An Organoselenium Compound Inhibits *Staphylococcus aureus* Biofilms on Hemodialysis Catheters In Vivo. Antimicrob. Agents Chemother..

[10] AlMojel N., AbdulAzees P.A., Lamb E.M., Amaechi B.T. (2023). Determining growth inhibition of *Candida albicans* biofilm on denture materials after application of an organoselenium-containing dental sealant. J. Prosthet. Dent..

[11] Jacobo U., Vopni R., Tran P., Patel S., Jain S., de Riese C.S., Reid T.W., de Riese W.T. (2022). Efficacy of organo-selenium-incorporated urinary catheter tubing for in vitro growth inhibition of *E. coli*, *K. pneumoniae*, *P. aeruginosa*, and *H. influenzae*. Int. Urol. Nephrol..

[12] McVerry B., Polasko A., Rao E., Haghniaz R., Chen D., He N., Ramos P., Hayashi J., Curson P., Wu C.Y. (2022). A Readily Scalable, Clinically Demonstrated, Antibiofouling Zwitterionic Surface Treatment for Implantable Medical Devices. Adv. Mater..

[13] Ahmed S.S., Shariq A., Alsalloom A.A., Babikir I.H., Alhomoud B.N. (2019). Uropathogens and their antimicrobial resistance patterns: Relationship with urinary tract infections. Int. J. Health Sci..

[14] Simmering J.E., Tang F., Cavanaugh J.E., Polgreen L.A., Polgreen P.M. (2017). The Increase in Hospitalizations for Urinary Tract Infections and the Associated Costs in the United States, 1998–2011. Open Forum Infect. Dis..

[15] Hammami F., Koubaa M., Chakroun A., Rekik K., Smaoui F., Elleuch E., Marrakchi C., Jemaa M.B. (2020). 1697. The Burden of Multidrug-Resistant Urinary Tract Infections. Open Forum Infect. Dis..

[16] Saint S., Lipsky B.A., Goold S.D. (2002). Indwelling Urinary Catheters: A One-Point Restraint?. Ann. Intern. Med..

[17] Smith P.W., Nicolle L.E. (2001). The Chronic Indwelling Catheter and Urinary Infection in Long-Term–Care Facility Residents. Infect. Control Hosp. Epidemiol..

[18] Palka M.A. (2014). Evidenced based review of recommendations addressing the frequency of changing long-term indwelling urinary catheters in older adults. Geriatr. Nurs..

[19] Pickard R., Lam T., MacLennan G., Starr K., Kilonzo M., McPherson G., Gillies K., McDonald A., Walton K., Buckley B. (2012). Antimicrobial catheters for reduction of symptomatic urinary tract infection in adults requiring short-term catheterisation in hospital: A multicentre randomised controlled trial. Lancet.

[20] Pickard R., Lam T., Maclennan G., Starr K., Kilonzo M., McPherson G., Gillies K., McDonald A., Walton K., Buckley B. (2012). Types of urethral catheter for reducing symptomatic urinary tract infections in hospitalised adults requiring short-term catheterisation: Multicentre randomised controlled trial and economic evaluation of antimicrobial- and antiseptic-impregnated urethral catheters (the CATHETER trial). Health Technol. Assess..

[21] So B., Kim J., Jo J.K., So H. (2024). Recent developments in preventing catheter-related infections based on biofilms: A comprehensive review. Biomicrofluidics.

[22] Fairweather-Tait S.J., Bao Y., Broadley M.R., Collings R., Ford D., Hesketh J.E., Hurst R. (2011). Selenium in Human Health and Disease. Antioxid. Redox Signal..

[23] Morgan S.D., Rigby D., Stickler D.J. (2009). A study of the structure of the crystalline bacterial biofilms that can encrust and block silver Foley catheters. Urol. Res..

[24] Desai D.G., Liao K.S., Cevallos M.E., Trautner B.W. (2010). Silver or nitrofurazone impregnation of urinary catheters has a minimal effect on uropathogen adherence. J. Urol..

[25] Srinivasan A., Karchmer T., Richards A., Song X., Perl T.M. (2006). A Prospective Trial of a Novel, Silicone-Based, Silver-Coated Foley Catheter for the Prevention of Nosocomial Urinary Tract Infections. Infect. Control Hosp. Epidemiol..

[26] Gauhar V., Castellani D., Teoh J.Y.C., Nedbal C., Chiacchio G., Gabrielson A.T., Heldwein F.L., Wroclawski M.L., de la Rosette J., Donalisio da Silva R. (2022). Catheter-Associated Urinary Infections and Consequences of Using Coated versus Non-Coated Urethral Catheters-Outcomes of a Systematic Review and Meta-Analysis of Randomized Trials. J. Clin. Med..

[27] Nicolle L.E. (2014). Catheter associated urinary tract infections. Antimicrob. Resist. Infect. Control.

[28] Weiner L.M., Webb A.K., Limbago B., Dudeck M.A., Patel J., Kallen A.J., Edwards J.R., Sievert D.M. (2016). Antimicrobial-Resistant Pathogens Associated with Healthcare-Associated Infections: Summary of Data Reported to the National Healthcare Safety Network at the Centers for Disease Control and Prevention, 2011-2014. Infect. Control Hosp. Epidemiol..

[29] Armbruster C.E., Smith S.N., Yep A., Mobley H.L.T. (2014). Increased incidence of urolithiasis and bacteremia during *Proteus mirabilis* and *Providencia stuartii* coinfection due to synergistic induction of urease activity. J. Infect. Dis..

[30] Vercellino T., Tran P., Reid T., Hamood A., Morse A. (2013). Evaluation of polymerized organo-selenium feed spacers to inhibit *S. aureus* and *E. coli* biofilm development in reverse osmosis systems. Desalination.

[31] Walker J.N., Flores-Mireles A.L., Pinkner C.L., Schreiber H.L., Joens M.S., Park A.M., Potretzke A.M., Bauman T.M., Pinkner J.S., Fitzpatrick J.A. (2017). Catheterization alters bladder ecology to potentiate *Staphylococcus aureus* infection of the urinary tract. Proc. Natl. Acad. Sci. USA.

[32] Tran P.A., O’Brien-Simpson N., Palmer J.A., Bock N., Reynolds E.C., Webster T.J., Deva A., Morrison W.A., O’Connor A.J. (2019). Selenium nanoparticles as anti-infective implant coatings for trauma orthopedics against methicillin-resistant *Staphylococcus aureus* and epidermidis: In vitro and in vivo assessment. Int. J. Nanomed..

[33] Navarro S., Sherman E., Colmer-Hamood J.A., Nelius T., Myntti M., Hamood A.N. (2022). Urinary Catheters Coated with a Novel Biofilm Preventative Agent Inhibit Biofilm Development by Diverse Bacterial Uropathogens. Antibiotics.

